# Supplementary material for: Pre-competition mental energy and performance relationships among physically disabled table tennis players
Source: PeerJ. 2022 Apr 14;10:e13294. doi: 10.7717/peerj.13294 (PMC9013477; doi:10.7717/peerj.13294)
Supplement: Supplemental Information 3 [file peerj-10-13294-s003.docx]

Code Book

Project title: Pre-Competition Mental Energy and Performance Relationships

Among Physically Disabled Table Tennis Players

Study #1

# Column 1: number of participants

# Column 2: gender (1= males; 2= females)

# Column 3: age

# Column 4: body weight

# Column 5: body height

# Column 6: year of sport participation

# Column 7: daily training hour

# Column 8: training time per week

# Column 9: order of competition

# Column 10-27: (AMES 1-18)

# Column 28: pre-competition mental energy total score

# Column 29: vigor, subscale of AMES

# Column 30: confidence, subscale of AMES

# Column 31: motivation, subscale of AMES

# Column 32: concentration, subscale of AMES

# Column 33: tireless, subscale of AMES

# Column 34: calm, subscale of AMES

# Column 35: subjective performance score

Study #2

# Column 1: number of participants

# Column 2: gender (1= males; 2= females)

# Column 3: age

# Column 4: body height

# Column 5: body weight

# Column 6: year of sport participation

# Column 7: daily training hour

# Column 8-26: (AMES 1-18)

# Column 27: pre-competition mental energy total score

# Column 28: subjective performance score

# Column 29: vigor, subscale of AMES

# Column 30: confidence, subscale of AMES

# Column 31: motivation, subscale of AMES

# Column 32: concentration, subscale of AMES

# Column 33: tireless, subscale of AMES

# Column 34: calm, subscale of AMES
